# Remaining Useful Life Prediction of Rolling Bearings Based on ECA-CAE and Autoformer

**DOI:** 10.3390/biomimetics9010040

**Published:** 2024-01-09

**Authors:** Jianhua Zhong, Huying Li, Yuquan Chen, Cong Huang, Shuncong Zhong, Haibin Geng

**Affiliations:** 1 College of Mechanical Engineering and Automation, Fuzhou University, Fuzhou 350108, China; jhzhong@fzu.edu.cn (J.Z.); 210220098@fzu.edu.cn (H.L.); chenyq118@163.com (Y.C.); hc18856362075@163.com (C.H.); genghb@fzu.edu.cn (H.G.); 2 Fujian Provincial Key Laboratory of Terahertz Functional Devices and Intelligent Sensing, Fuzhou University, Fuzhou 350108, China

**Keywords:** deep learning, rolling bearings, Autoformer

## Abstract

In response to the need for multiple complete bearing degradation datasets in traditional deep learning networks to predict the impact on individual bearings, a novel deep learning-based rolling bearing remaining life prediction method is proposed in the absence of fully degraded bearng data. This method involves processing the raw vibration data through Channel-wise Attention Encoder (CAE) from the Encoder-Channel Attention (ECA), extracting features related to mutual correlation and relevance, selecting the desired characteristics, and incorporating the selected features into the constructed Autoformer-based time prediction model to forecast the degradation trend of bearings’ remaining time. The feature extraction method proposed in this approach outperforms CAE and multilayer perceptual-Attention Encoder in terms of feature extraction capabilities, resulting in reductions of 0.0059 and 0.0402 in mean square error, respectively. Additionally, the indirect prediction approach for the degradation trend of the target bearing demonstrates higher accuracy compared to Informer and Transformer models, with mean square error reductions of 0.3352 and 0.1174, respectively. This suggests that the combined deep learning model proposed in this paper for predicting rolling bearing life may be a more effective life prediction method deserving further research and application.

## 1. Introduction

The complexity and sophistication of the structure of modern mechanical operating equipment has been further enhanced by the progress and continuous improvement of the level of technology and production processes. However, due to the combined effect of external environment and internal equipment, the actual performance and health condition of rotating equipment will inevitably show a tendency of decline. If this tendency of degradation reaches a certain threshold, the equipment will not be able to complete the tasks and functions it is responsible for, and if it continues to operate, it will lead to the damage of its neighbouring parts and paralysis of the equipment, causing immeasurable property damage and threatening the staff. Life safety Rolling bearings, as important components of rotating equipment, are subject to wear, deformation and dislodgement during continuous operation. Research results show that bearings cause more than 30% of rotating equipment failures [1,2,3]. Therefore, predictive maintenance of rotating equipment can be achieved by studying rolling bearings. In recent years, life prediction techniques have developed rapidly. The existing life prediction methods can be broadly classified into physical failure model-based methods and data-driven methods [4,5,6,7]. For complex, obtaining the physical failure mechanism of highly reliable equipment is time-consuming and difficult. In contrast, data-driven prediction methods do not rely on the failure mechanism of the equipment, but they require monitoring the operation of the equipment and collecting valid failure data or performance degradation data [8,9,10]. However, it requires monitoring of the equipment operation and collecting valid failure data or performance degradation data. Data-driven approaches generally include those based on statistically driven models and reliability functions, as well as those based on machine learning and deep learning models are the main directions of current life prediction research [11,12,13,14]. For rolling bearings, the main prediction steps include: (1) extracting failure characteristics and degradation curves using signal processing or machine learning; (2) constructing health indicators using deep learning or degradation function models to characterize life thresholds; (3) using the trained deep learning degradation models for life prediction or model fitting the obtained degradation curves to finally obtain the RUL [15,16,17]. Li Hailang et al. combined the trend consistency constraint and parallel variance constraint to extract features from convolutional self-coding to reduce the individual differences of bearings with the same tag features, which improved the bearing prediction accuracy to a certain extent [18,19]. Zhang Guangyu used convolutional autoencoders to learn deep features from data and solve the problem of lack of labeled data in the actual operation of mechanical equipment. The optimized deep feature data is fused with the original data and input into the gated recurrent unit for temporal feature extraction, addressing issues such as insufficient utilization of temporal features in the data and achieving remaining useful life prediction [20].

She Daoming proposed a novel method combining deep autoencoders and minimum quantization error to accurately describe the dynamic degradation process of rolling bearings. The results showed that the trend value, monotonicity value, robustness value, and fusion evaluation criterion value of the health indicators constructed using this method were all higher than those of single-layer autoencoder models and traditional Principal Component Analysis dimensionality reduction methods [21].

These researchers applied convolutional autoencoder techniques to address the issues of unlabeled data and utilization of temporal features, effectively improving the accuracy of remaining useful life prediction for bearings. Their proposed methods outperformed traditional methods in terms of accuracy and evaluation criteria.

Statistically driven model and reliability function are applied for RUL prediction, such as Li NaiPeng et al. [22]. A general Wiener process model included the multi-source observation function and mapping function is used to describe the causal and correlation relationships between the state and the data, and a particle filtering algorithm is also applied to dynamically match the multi-sensor data with the model to predict the RUL. This method disposes the problems of deep learning algorithms over-relying on training data and lacking necessary empirical guidelines, but further discussion is needed due to the large amount of data required and the weight assignment of multi-sensor data. Machine learning SVM was first proposed by Cortes and Vapnik in 1995 to solve classification and regression problems for analyzing small samples and multi-dimensional data [23]. Chen et al. [24] used SVM to predict the RUL of an aero-engine and to predict the RUL of an operating equipment based on the improved similarity theory combined with the RUL results of the engine. This method has a high degree of process visualization, but is more complex, containing a priori knowledge of data processing, feature screening, parameter optimization, etc., and also requires a large amount of data for training, which makes it difficult to achieve prediction in a practical working environment.

Wang et al. [25] proposed a health indicator generation network model based on spatial convolutional long and short term memory neural network (ConvLSTM), which directly mines the features reflecting the degradation degree from the collected raw signals to construct health indicators and achieve the output of RUL expectancy results. The health indicators constructed by this method have better trending, monotonicity and robustness, while the accuracy of RUL prediction is higher. However, the model aims to stack the network and improve the feature extraction capability, but no specific explanation can be made as well as a large amount of data is required as the training set, and different training sets and training processes can cause large fluctuations in the model. Qiao Xiandong and others used the Autoformer model to train temperature data. Compared to other Transformer models, Autoformer has a lower error rate and higher efficiency, which improves the accuracy of temperature forecasting [26]. Wu Haixu and others addressed the long-term prediction problem of time series by designing Autoformer as a novel decomposition architecture with autocorrelation mechanism. As a result, Autoformer achieved state-of-the-art accuracy on six benchmarks [27].

The essence of both deep learning and machine learning is feature extraction. The difference is that deep learning is feature extraction from multiple levels and angles of the original data through neural nodes, while machine learning is data decomposition through functions, which explains why deep learning has weaker interpretability but relatively better results. The main challenges of current bearing life prediction research can be summarized as follows: 1. Scarcity and imbalance of bearing fault data: traditional deep learning requires a large amount of complete degraded bearing data for model training. 2. Characteristic selection and extraction: the feature extraction method needs to be improved, and specific explanations cannot be made. In order to increase the number of samples, Yanfang Fu et al. [28] trained an improved deep residual network (SE-ResNet18) fault diagnosis model based on channel attention mechanism on the basis of the existing fault samples by using Wavelet Packet Decomposition (WPD) denoising and Conditional Variational Autoencoder (CVAE), and the enhanced fault samples improved the accuracy of fault diagnosis. Dominik Łuczak et al. [29,30] solved the multi-classification problem by converting the acquired one-dimensional signal into RGB image data and training a convolutional neural network with the image based on the problem of feature selection and extraction, and Min Su Kim [31] efficiently extracted the feature map of each X, Y, and Z axis from the three-axis vibration signal by grouping the one-dimensional convolutions, and the feature map extracted from each axis consisted of a specific frequency of each axis without domain transformation to train the end-to-end model. The model classifies faults based on the frequency characteristics of each axis. Liang et al. [32] used a one-dimensional dilated convolutional neural network to realize the feature extraction and fault mode classification of the excitation current, and further used the Score-CAM activation mapping algorithm to analyze the diagnostic mechanism of the model, taking into account the accuracy and interpretability of the model.

In summary, in order to minimize the impact of limited degraded bearing data on the accuracy of life prediction, this paper proposes a new deep learning network model framework. This framework processes the extracted features with higher interpretability, introduces a new feature extraction method called ECA-CAE, and selects features with better linear and exponential trends. This method has better feature extraction capabilities than CAE and MLP-AE. The Autoformer model is used for degradation trend prediction and combined with a double exponential model for RUL prediction [33]. Furthermore, in terms of deep learning, a single bearing prediction approach is adopted, where the first half is used for training and the second half is for prediction. Compared with traditional deep learning methods that require a large amount of complete degraded bearing data for model training, this method only uses the first half of the current bearing degradation features to predict future degradation trends. This solves the problem of non-optimal model parameters due to insufficient data volume. By comparing with the latest Informer and Transformer models [34,35], the advantages of the proposed method are validated. Additionally, this model is suitable for predicting the life of bearings under the same operating conditions. Based on this, the next degradation trend can be predicted, and corresponding measures can be formulated to mitigate degradation.

## 2. Principle Introduction

### 2.1. CAE Model

**Figure 1:**
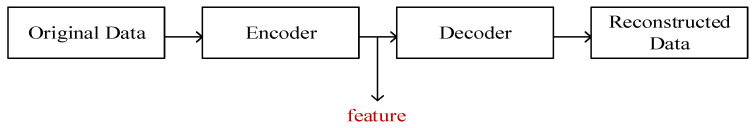
The working principle of the CAE model.

Auto-encoders are neural networks designed to replicate their input to the output. They work by compressing the input into a latent-space representation and then reconstructing the output of this representation, the more the output converges to the input, the more the features extracted by the network represent the internal features of the input data. This network consists of two parts, Encoder and Decoder. Figure 1 shows the implementation process of this network.

Convolutional auto-encoder is a convolutional network that replaces the fully-connected layer. The encoder consists of convolutional and pooling layers, and the decoder consists of an inverse convolutional layer.

In the encoder, *k* convolutional kernels (*W*) are initialized, and each convolutional kernel is paired with a bias b to generate k features h after convolution with the input x. The activation function is sigmoid. equation is as follows:

(1) $$h_{k}=\sigma (x\times W^{k}+b^{k})$$

Pooling operation (Max Pooling): When pooling the features generated above, the matrix of the position relationship at the time of pooling should be retained to facilitate the operation of inverse pooling later.

In the decoder, the inverse pooling operation is performed on the features generated above, and a matrix that preserves the positional relationships at the time of pooling is used to restore the data to the corresponding positions of the matrix of the original size.

The transpose of each feature h with its corresponding convolution kernel The convolution operation is performed and the result is summed, then the bias c is added and the activation function remains sigmoid. equation is as follows:

(2) $$y=\sigma (\sum_{k}h^{k}\times W^{\sim k}+c)$$

In order to make the network better, the weights are updated and the minimum mean squared error (MSE) function is used, i.e.,: the target value minus the squared sum of the actual values and then the mean value, 2n is used to simplify the derivation. The formula is as follows:

(3) $$E(\theta )=\frac{1}{2n}\sum_{i=1}^{n}{\left(x_{i}-y_{i}\right)}^{2}$$

### 2.2. ECANet

**Figure 2:**
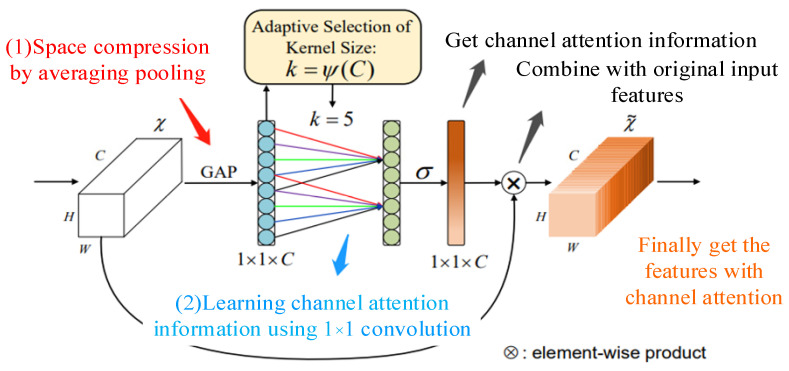
ECA network.

To cope with the problem of weight assignment among different channels, scholars have proposed ECANet, ECA can assign different weights to multiple channels of the input without changing the input feature size, and the structure of ECA is as shown in Figure 2:

The flow of the ECA model is as follows.

(1) Features with input dimensions H × W × C;
(2) Compressing the input features using global average pooling to obtain 1 × 1 × C features;
(3) The resulting features are subjected to channel learning using 1 × 1 convolution to learn the importance between different channels and to assign weights;
(4) Finally, the feature map 1 × 1 × C with weights is combined with the input features H × W × C to obtain the output features with channel attention.

In doing the convolution process, dynamic convolution kernels are used to effectively extract features under different sensory fields and learn the weights between different channels. Dynamic convolution kernel means using an adaptive function to decide the convolution kernel size; the size of the convolution kernel is determined by the number of channels; the larger the number of channels, the larger the convolution kernel and the stronger the cross-channel interaction; on the contrary, in layers with smaller number of channels, smaller convolution kernels are used and less cross-channel interaction is performed; the adaptive function is as follows:

(4) $$k=\Psi (C)={\left|\frac{\log_{2}(C)}{\gamma }+\frac{b}{\gamma }\right|}_{odd}$$

where k represents the size of the convolution kernel; C represents the number of channels; indicates that k can only take odd numbers; and b is set to 2 and 1, which is used to change the ratio between the number of channels C and the convolution kernel.

### 2.3. Autoformer Model

With the good performance of Transformer models on natural language (NLP) processing, researchers have turned their attention to temporal processing, however, temporal processing suffers from the following problems: as the prediction time lengthens, it is difficult to find reliable temporal dependencies from complex temporal patterns by directly using the self-attention mechanism; secondly, due to the self-attention secondary complexity problem, the model has to use its sparse version, but it will limit the information utilization efficiency and affect the prediction effect.

**Figure 3:**
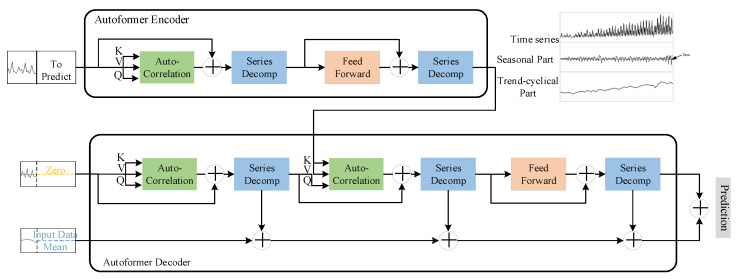
Autoformer Network.

The Autoformer model proposes a progressive sequence decomposition and autocorrelation mechanism to solve the above problems, and the model is shown in Figure 3, in which the encoding and decoding parts include an autocorrelation module, a sequence decomposition module, and a feedforward neural network. The feed-forward temporal network acts as a deeper feature extraction.

The series decomposition module (series decomposition) is based on the idea of sliding average, smoothing the periodic terms and highlighting the trend terms:

(5) $$\chi_{t}=AvgPool(Padding(\chi ))$$

(6) $$\chi_{s}=\chi -\chi_{t}$$

where χ is the hidden variable to be decomposed, $\chi_{t}$ and $\chi_{s}$ are the trend and period terms.

In the Encoder part, a stepwise elimination of the trend term is taken to obtain the periodic terms $S_{en}^{l,1}$, $S_{en}^{l,2}$. And based on this periodicity, an autocorrelation mechanism is used to aggregate similar sub processes of different periods:

(7) $$S_{en}^{l,1},\_=SeriesDecomp(AutoCorrelation(\chi_{en}^{l-1})+\chi_{en}^{l-1})$$

(8) $$S_{en}^{l,2},\_=SeriesDecomp(FeedForward(S_{en}^{l,1})+S_{en}^{l,1})$$

In the Decoder section, the trend term and the period term are modeled separately. In which, for the period term, the autocorrelation mechanism uses the periodic nature of the sequence to aggregate subsequences with similar processes in different cycles; for the trend term T, the trend information is gradually extracted from the predicted hidden variables using a cumulative approach.

(9) $$S_{de}^{l,1},T_{de}^{l,1}=SeriesDecomp(AutoCorrelation(\chi_{de}^{l-1})+\chi_{de}^{l-1})$$

(10) $$S_{de}^{l,2},T_{de}^{l,2}=SeriesDecomp(AutoCorrelation(S_{de}^{l,1},\;\chi_{en}^{N})+S_{de}^{l,1})$$

(11) $$S_{de}^{l,3},T_{de}^{l,3}=SeriesDecomp(FeedForward(S_{de}^{l,2})+S_{de}^{l,2})$$

(12) $$T_{de}^{l}=T_{de}^{l-1}+W_{l,1}\times T_{de}^{l,1}+W_{l,2}\times T_{de}^{l,2}+W_{l,3}\times T_{de}^{l,3}$$

Based on the above progressive decomposition architecture, the model can gradually decompose the hidden variables in the forecasting process and obtain the forecasting results of cycle and trend components respectively through the autocorrelation mechanism and accumulation, so as to realize the alternate and mutual promotion of decomposition and optimization of forecasting results.

## 3. Feature Extraction Method and Remaining Useful Life Prediction Algorithm

### 3.1. ECA-CAE

**Figure 4:**
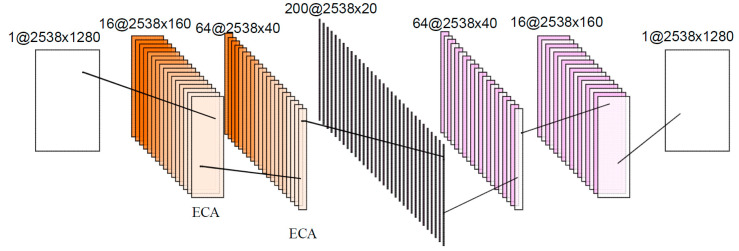
ECA-CAE network.

Combining the advantages of different levels and fields of view of convolutional networks and the weighting mechanism of channel attention mechanism, this paper-proposes a new feature extraction method: ECA-CAE. the specific network structure is shown in Figure 4. Table 1 describes the parameter settings of the feature extraction model. The channel attention mechanism introduces the weights into each channel.

In order to verify the effectiveness of the ECA-CAE method proposed in this chapter, the proposed method is compared with the ECA encoder MLP and the convolutional self-encoder CAE to verify the effectiveness of the ECA-CAE based method proposed in this chapter, Table 2 shows the comparison of the encoder effect of the three methods, the smaller the MSE and MAE, the lower the loss of new data generated by the encoder and the closer to the actual data.

**Figure 5:**
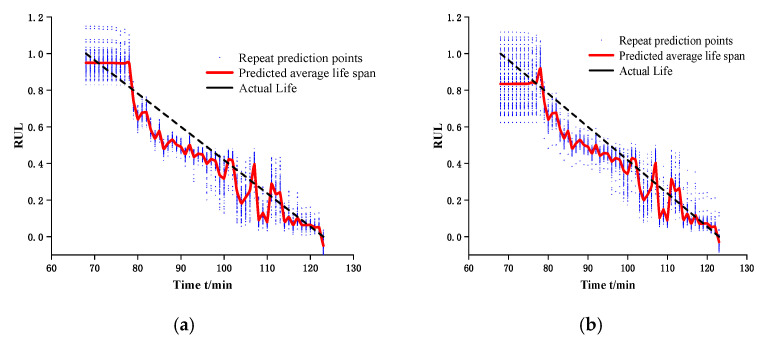
Remaining useful life prediction. (**a**) the results of the proposed method in this chapter. (**b**) the prediction results under CAE network.

In order to verify the effectiveness of the extracted features, bearings 1-1 and 1-2 (the degradation curve of this bearing is basically the same, and the prediction effect is better under single-bearing training) are taken, and three one-dimensional convolutional networks (32@8 × 1), (64@4 × 1), (128@4 × 1) and a fully connected layer are constructed. The 200 × 1 features proposed in this chapter and the features proposed in the CAE network are used to predict the remaining life, where the training and prediction data are both in the failure stage, and the experiment is repeated 100 times to take the average value. The prediction results are shown in Figure 5. The right panel shows the prediction results under CAE network, and the left panel shows the results of the proposed method in this chapter. In 100 replicate experiments, the fluctuations of the prediction results are smaller compared to CAE, i.e., the prediction interval is smaller, while Table 3 shows that the proposed method is smaller in terms of the error with the true lifetime.

### 3.2. Remaining Useful Life Prediction Based on ECA-CAE and Autoformer Model

**Figure 6:**
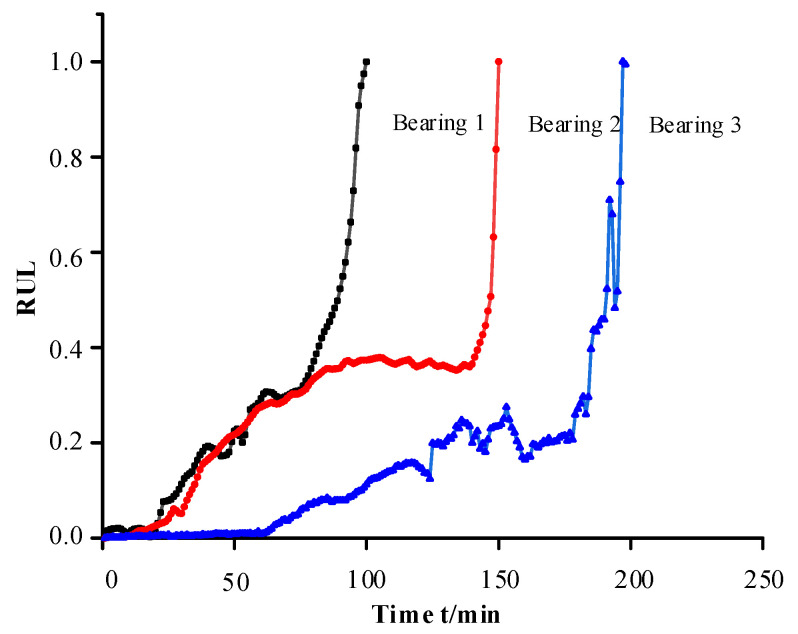
Degradation trend of different bearings.

The problems encountered in traditional deep learning of multiple bearings to predict single bearings include the unification of thresholds and the trend differences among multiple bearings. During the training of the network model, due to the difference in the failure criteria adopted by different bearings or the sampling time interval is too large, there is easily too much difference in the vibration signals leading to the threshold value cannot be unified; the trend difference problem is shown in Figure 6, if bearing 1,2 is used as training, the model is unable to learn any degradation process about bearing 3, and the RUL of bearing 3 is predicted on this basis, without deliberate tuning of the parameter optimization, the theoretical accuracy is not high.

**Figure 7:**
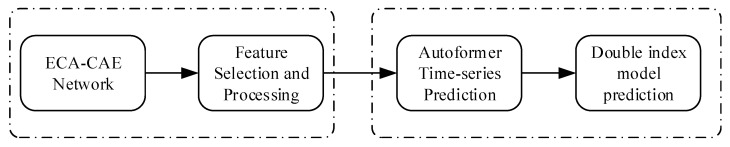
Flow of the method in this paper.

The RUL prediction among individual bearings proposed in this paper is a time series prediction of the remaining degradation trend of the bearings, based on which a double exponential model is fitted to achieve the life prediction. The processing is shown with reference to Figure 7, and the model consists of two modules: signal processing and life prediction.

After the original vibration signal is processed by the encoder of the convolutional self-encoder, the obtained data is 200 × 20. In order to preserve the spatial information and minimize the loss of decoding, the global average pooling layer (GAP) is used outside the encoder to obtain 200 × 1 data. Using GAP instead of fully connected layer (FC) will reduce a lot of training time, reduce the spatial parameters to make the model more robust, and at the same time anti-overfitting effect is better.

The 200 dimensional signals obtained by ECA-CAE have similar features and useless features with small variance, which need to do feature processing. Variance Threshold is to filter out the features with no trend or relatively flat trend, which are not enough to represent the degradation characteristics. threshold is the most important parameter in variance filtering, the parameter will filter out the useful features, the parameter is taken as 0.1. F-test can be used to capture the linear relationship between features and rank them according to the size of linear relationship. Similar to the F-test, the mutual information method can capture the nonlinear relationships from among the features. The feature filtering relationships are shown in Table 4.

The Autoformer model used in this study for lifespan prediction is a type of time series forecasting model. It achieves progressive decomposition of time series by embedding sequence decomposition as an internal unit into the encoder-decoder architecture. In the Autoformer model, there are some hyperparameters that can potentially impact model performance: During the hyperparameter optimization process, choosing ‘Gelu’ as the activation function is preferred for better numerical stability compared to ‘Relu’ or ‘Sigmoid’. The choice of the number of iterations takes into account the trade-off between training time and training quality, and it was found that around 10 iterations yield better performance. Different regularization parameter values were used to train multiple models, and their performance was evaluated on the validation set. Eventually, the regularization parameter value of 0.05, which performed best on the validation set, was selected. Furthermore, a larger learning rate can accelerate training, while a smaller learning rate can make the model more stable. The optimal learning rate is determined to be 0.0001.

**Figure 8:**
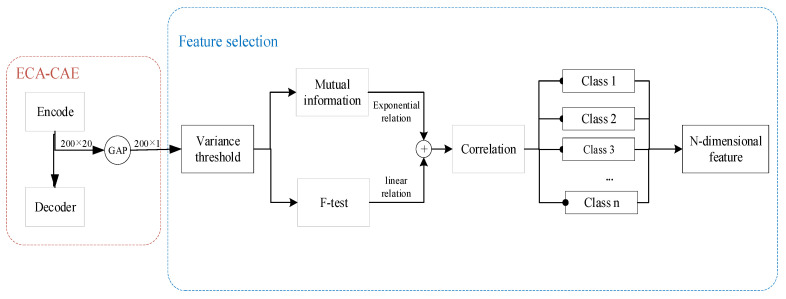
Feature processing process.

As shown in Figure 8, the 200-dimensional data is obtained after global average pooling, and the features with small variance are first filtered by variance filtering; then the features with obvious exponential and linear trends are screened out; the correlation between the features is analyzed, the features are grouped by correlation, and the features with high robustness and monotonicity in each category are screened out. Finally, the n-dimensional features with exponential and linear trends are obtained, which also best represent the features extracted by the 200-dimensional CAE network.

## 4. Experimental Validation

### 4.1. Data Sources

This paper uses the bearing data from Xi’an Jiaotong University as the original data [25]. The dataset provides diverse types of working conditions and clearly lists the fault location of each failed bearing with high frequency resolution on the basis of giving the whole life cycle vibration data, which makes the application scenario of the dataset broadened and can be widely used for health monitoring, fault diagnosis and RUL prediction of mechanical equipment.

**Figure 9:**
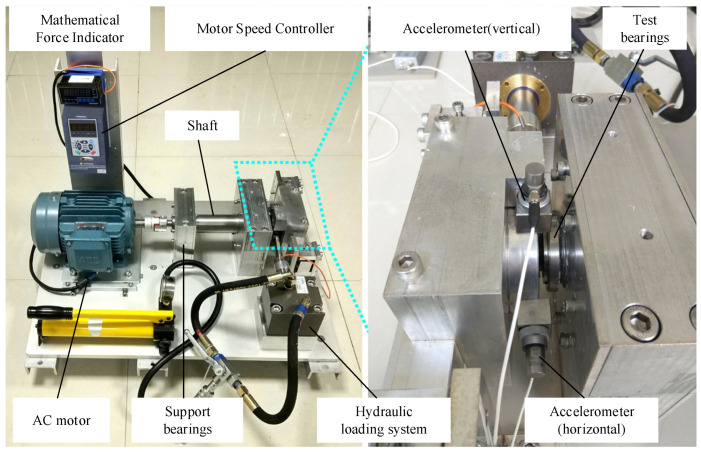
Bearing acceleration life test bench.

The experimental bearing accelerated life testing platform is shown in Figure 9. The tested bearing is an LDK UER204 rolling bearing. Three types of operating conditions were designed for the experiment, as shown in Table 5, with five bearings under each operating condition. Table 6 provides detailed information for each test bearing, including its corresponding operating condition, total number of data samples, basic rated life (L10), actual life, and failure location.

**Figure 10:**
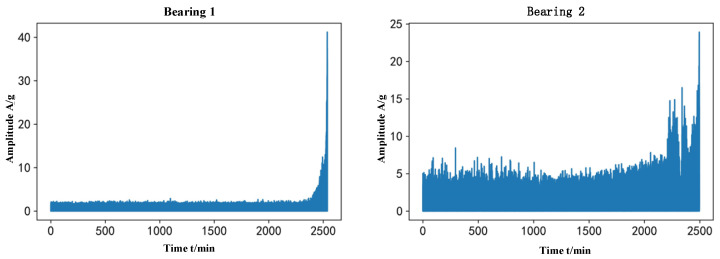
Original vibration signal trend.

The sampling frequency of this experiment is 25.6 kHz, the sampling interval is 1 min, the length of each sample is 1.28 s, and saved as a csv file, the number of samples N represents the bearing life for N min. In this paper, take bearing 1 under working condition three as an example, the number of samples is 2538, the horizontal vibration signal under each sample is equally spaced out 1280 points, and the trend of the original vibration signal is shown in Figure 10: 

### 4.2. Data Processing and Partitioning

**Figure 11:**
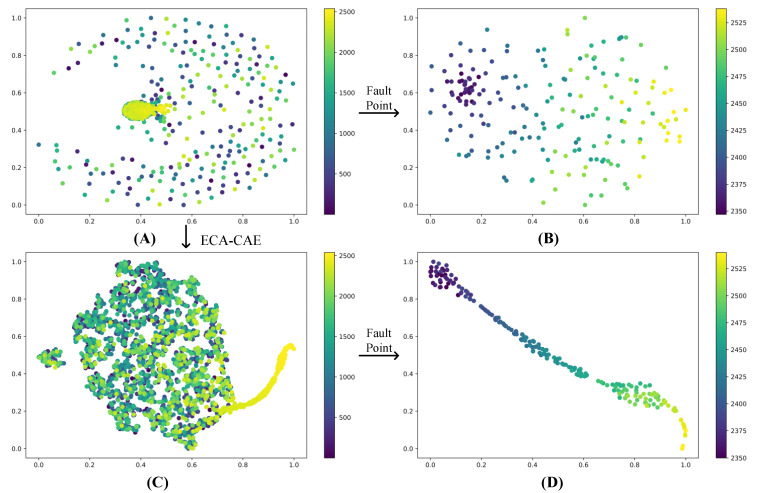
Feature visualization. (**A**) Data not processed by CAE. (**B**) A portion of the A data after dimensionality reduction by T-SNE. (**C**) Data processed by CAE. (**D**) A portion of the C data after dimensionality reduction by T-SNE.

Take bearing 1 as an example, using 2.1 signal processing process, after getting the data of 2538 × 200, the data will be made T-SNE processing, the purpose is to separate the fault stage and normal stage, the signal of normal stage is smooth, and it has no guiding meaning to the actual prediction. In Figure 11, A and B are the data without CAE processing, and C and D are the data after CAE processing, where B and D are part of the data of A and C respectively taken out separately for T-SNE. the distribution of fault data points in B is scattered and the trend is not obvious. the trend of the fault phase in D is more obvious, which shows that the data processing method of this paper extracts the effective degradation characteristics.

**Figure 12:**
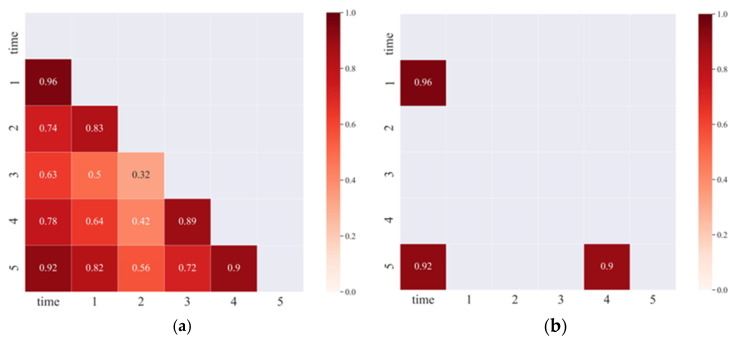
Correlation analysis between features. (**a**) The correlation between the features. (**b**) The correlation with time.

Since the signal distribution of the normal operation phase of the bearing is irregular, the features related to the life time cannot be extracted, so the failure phase data is chosen as the training and prediction data. After feature screening, five features representing different degradation characteristics were finally obtained, and the correlation between the features and the correlation with time (time) were calculated as shown in Figure 12. The correlation coefficients among the screened features were less than 0.9, which verified that the five features had different degradation curves, and proved that the classification effect of the proposed method was good, and the screened features were used to represent 200 features.

**Figure 13:**
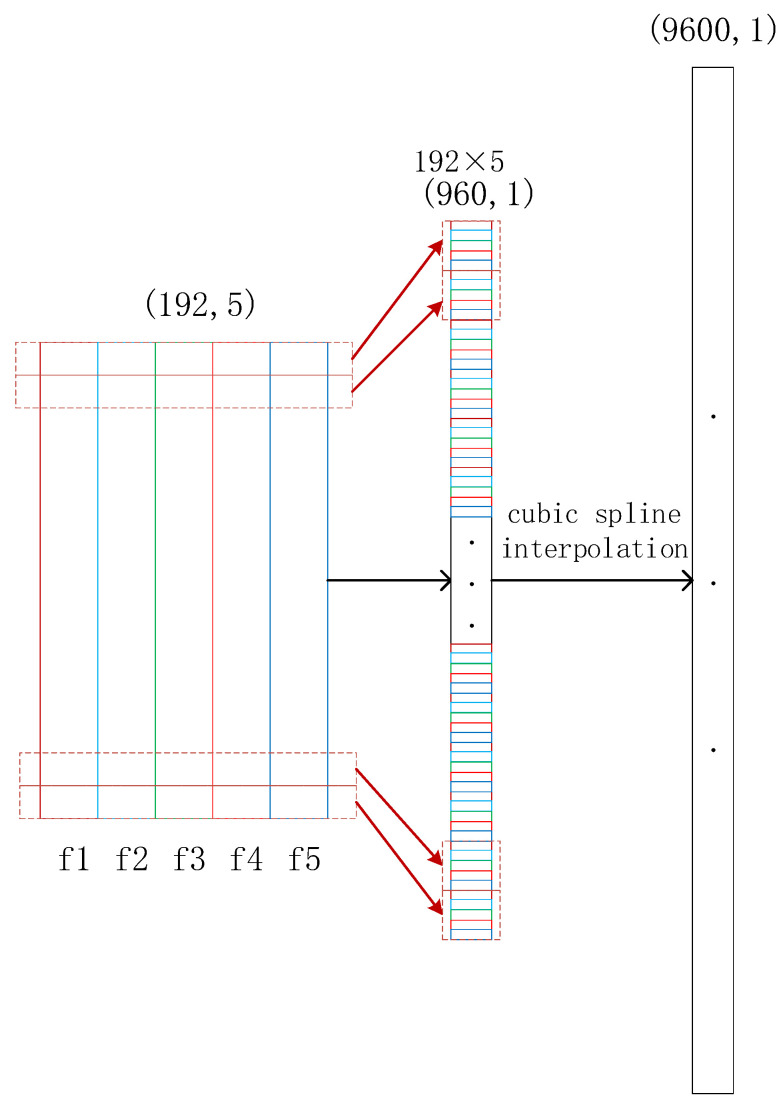
Sequence splicing.

If the resulting features are considered for lifetime prediction at this point, the required sequence length is not sufficient to support the training of the model, which leads to overfitting. At the same time, if the period information is added, the prediction accuracy of the sequences will be greatly increased. Therefore, a multi-dimensional sequence splicing approach is proposed as shown in Figure 13 below:

In the figure, f1 to f5 are 5 features, each feature is 192 in length, and after feature splicing, a sequence of length 192 × 5 is obtained. To achieve a smooth connection between the points of the sequence, the sequence is uniformly increased to a length of 9600 using cubic spline interpolation.

**Figure 14:**
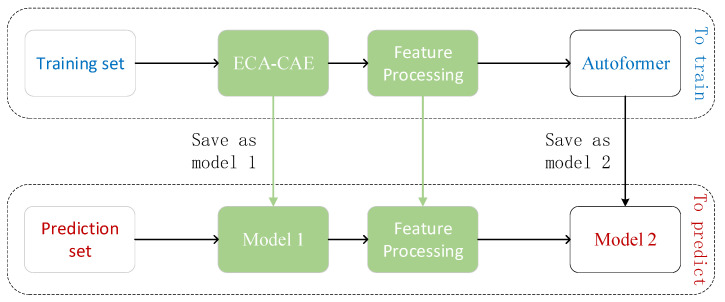
Model operation framework.

The first 70% of the failed data points are used as training data, 10% as validation, and the remaining data as prediction. To avoid data leakage, the first 70% of the fault stage data is first used as data input to the ECA-CAE model (the saved model), and after data filtering and splicing, it is input to the Autoformer model as the training set; the validation and prediction sets use the remaining 30% of the data, and this data set is input to the saved CAE model to realize the prediction of the sequence in Model 2. See Figure 14 for the specific process.

### 4.3. Timing and Remaining Useful Life Prediction

**Figure 15:**
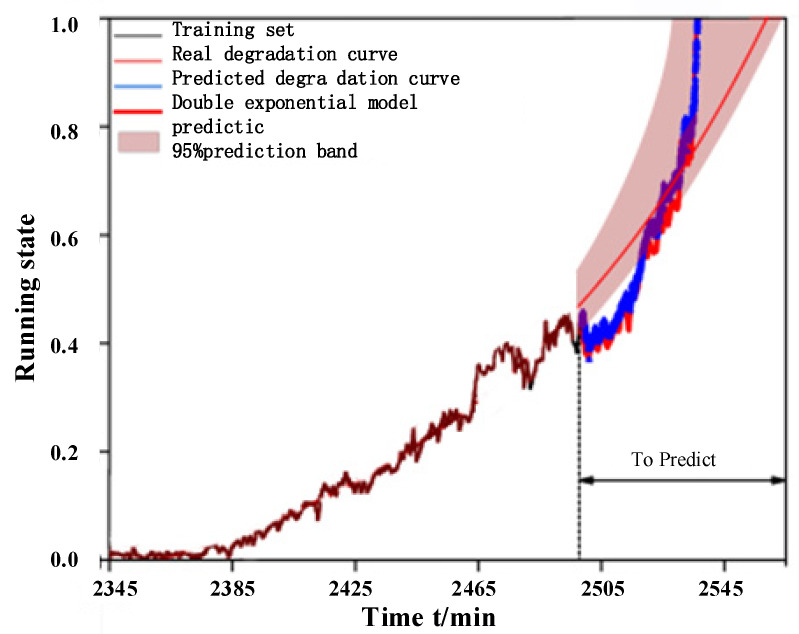
Bearing 1 Prediction and Fitting.

The above-mentioned training set and prediction set are processed separately, meanwhile, the input length of Autoformer model is 200 and the prediction length is 50, and the prediction result is used as the next input, the parameter settings of this model are shown in Table 7, and the final results obtained are shown in Figure 15: If the prediction is taken to the training set by fitting the double exponential model, the error exists between the fitted curve and the actual degradation curve because the degradation trend at the later stage is unknown, and the proposed method in this paper is the prediction of the degradation process, and the prediction results tend to be close to the real life.

**Figure 16:**
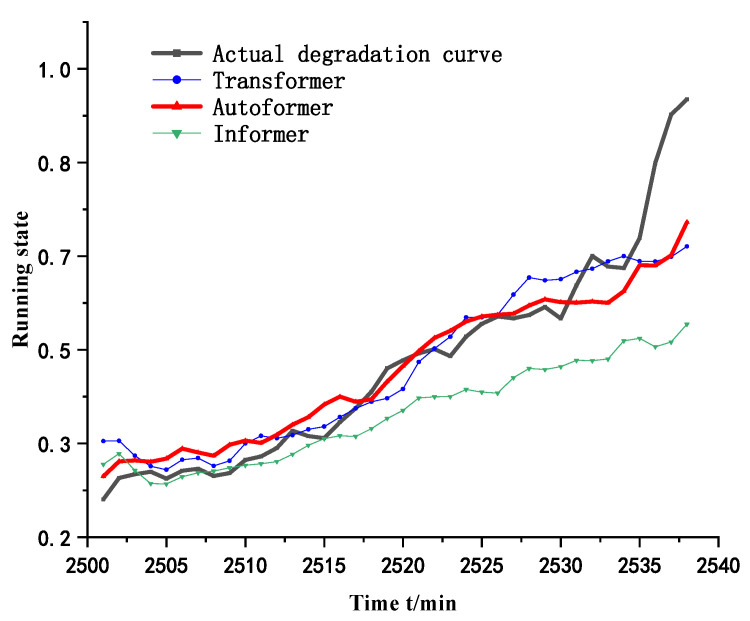
Prediction curves of the three methods.

In order to verify the effectiveness of the proposed method, it is compared with Transformer and Informer models respectively, and the data processing and model parameters are taken to be consistent with the proposed method in this paper in order to ensure the experimental results. Figure 16 shows the time series prediction curves and the results of the double exponential fitting under different models.

Table 8 shows the comparison of the errors of different methods and the proposed method in this paper, and the results show that the proposed method has the smallest mean square error (MSE) and mean absolute error (MAE).

In order to verify the effectiveness of the proposed scheme, the scheme is compared with the performance characterization index of SVR, DBN, CNN, Bi-LSTM, etc. The results show that the performance of the proposed method is obviously due to the performance of each lightweight architecture. Detailed numerical values are shown in Table 9.

**Figure 17:**
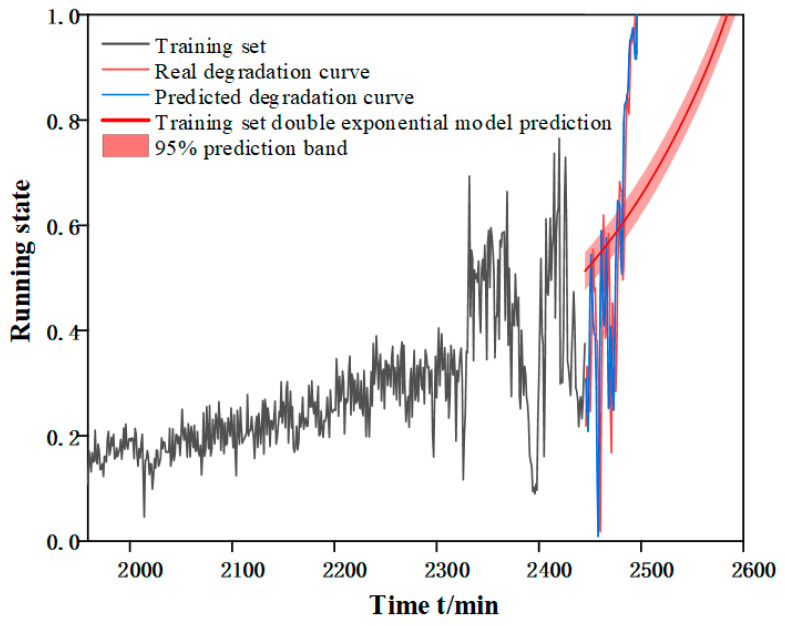
Prediction effect of bearing 2.

**Figure 18:**
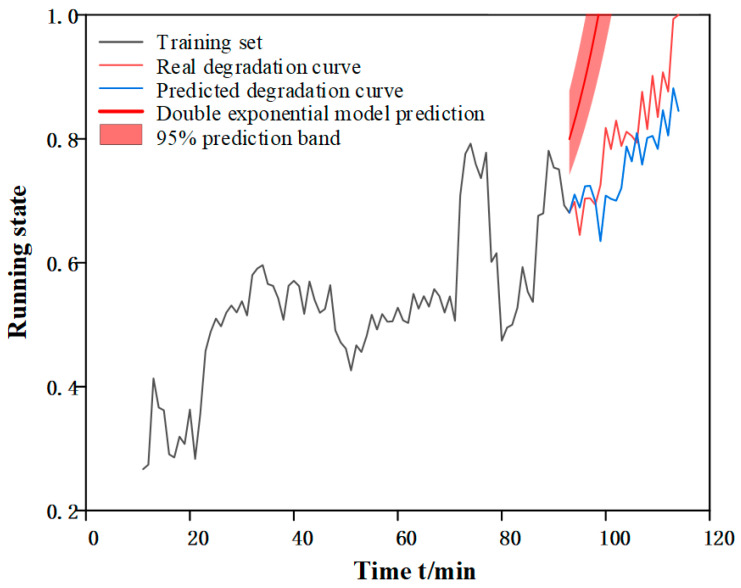
Prediction effect of bearing 5.

The main purpose of deep learning based network models, compared to machine learning, is to build an environment with autonomous judgment and prediction. Although the method proposed in this paper can make accurate prediction for a single bearing, the ultimate goal is to predict the RUL for a certain equipment under the same working condition or even different working conditions. For this reason, taking working condition three as an example, the model parameters of bearing 1 signal processing and feature screening are retained for the prediction of bearing 2 and bearing 5. Since the degradation curve of each bearing is different, so each bearing does a separate Autoformer model training, and the prediction results obtained are shown in Table 10, from which it can be seen that the method proposed in this chapter is significantly better than the double exponential model in terms of prediction accuracy. Figure 17 and Figure 18 shows the prediction results of the double exponential model for bearing 2 and bearing 5 and the ECA-CAE + Autoformer model proposed in this chapter.

## 5. Conclusions

To address the challenging problem of RUL (Remaining Useful Life) prediction for rolling bearings, a novel solution has been proposed: automatic time series forecasting to indirectly predict RUL based on degradation trends. The advantages of this method are as follows:

A novel feature extraction method, ECA-CAE, has been introduced, which outperforms CAE and MLP-AE in terms of feature extraction capabilities.

Under the premise of enhancing feature interpretability through Convolutional Autoencoders (CAE), this chapter presents a degradation trend prediction method based on individual bearings. In contrast to traditional deep learning methods that require a large amount of complete degradation bearing data for model training, this method only utilizes the first half of the current bearing’s degradation features to predict future degradation trends. Moreover, this model is suitable for predicting bearing life under the same operating conditions. Based on this, it predicts the next degradation trend and formulates corresponding measures to mitigate degradation. While ECA-CAE and Autoformer provide effective methods for predicting rolling bearing life, their effectiveness depends on various factors such as data quality, model complexity, interpretability, and generalization ability. When applying these methods in practical industrial scenarios, it is essential to carefully consider these limitations and strike a balance between model complexity and feasibility.

There is still significant research potential in the field of rolling bearing life prediction. Future research directions should focus on improving model performance, generalization ability, practicality, and interpretability. Research efforts should also explore how to integrate these models into real-time monitoring systems to achieve real-time health monitoring and maintenance of bearings. Additionally, considerations should be given to how to implement online learning and transfer learning to adapt to changes and new data in the bearing operating process, meeting the needs of predictive maintenance in the industrial sector.

## Figures and Tables

**Table 1:** Parameters of ECA-CAE model.

Parameter	Convolution Kernel Size	The Number of Convolution Kernels
Convolutional layer 1	1 × 8	1
Convolutional layer 2	1 × 4	16
Convolutional layer 3	1 × 2	64
Deconvolutional layer 1	1 × 2	64
Deconvolutional layer 2	1 × 4	16
Deconvolutional layer 3	1 × 8	1

**Table 2:** Comparison of losses of different encoders.

Name	MSE	MAE
MLP-AE	0.1013	0.2044
CAE	0.0670	0.1871
**ECA-CAE**	**0.0611**	**0.1626**

**Table 3:** Life prediction loss values.

Name	MSE	MAE
CAE + 1DCNN	0.1780	0.3504
**ECA-CAE + 1DCNN**	**0.1654**	**0.3321**

**Table 4:** Summary of filtering methods.

Name	Description	Parameters
Variance filtering	Input the variance threshold and return the new feature matrix with variance greater than the threshold	Pursue characteristics with large variance
F-test	Captures only linear correlations	Characteristics pursuing p less than the significance level
Mutual Information	Can capture any correlation	Pursuit of mutual information greater than 0 characteristics

**Table 5:** Bearing acceleration life test condition.

Condition Number	1	2	3
rotate speed/(r/min)	2100	2250	2400
Radial force/kN	12	11	10

**Table 6:** List of XJTU-SY bearing dataset information.

Working Conditions	Data Set	Total Number of Samples	L_10_	Actual Lifespan	Failure Location
1	Bearing1_1	123	5.600~9.677 h	2 h 3 min	Outer ring
	Bearing1_2	161		2 h 41 min	Outer ring
	Bearing1_3	158		2 h 38 min	Outer ring
	Bearing1_4	122		2 h 2 min	Cage
	Bearing1_5	52		52 min	Inner ring, Outer ring
2	Bearing2_1	491	6.786~11.726 h	8 h 11 min	Inner ring
	Bearing2_2	161		2 h 41 min	Outer ring
	Bearing2_3	533		8 h 53 min	Cage
	Bearing2_4	42		42 min	Outer ring
	Bearing2_5	339		5 h 39 min	Outer ring
3	Bearing3_1	2538	8.468~14.632 h	42 h 18 min	Outer ring
	Bearing3_2	2496		41 h 36 min	Inner ring, Rolling body, Cage, Outer ring
	Bearing3_3	371		6 h 11 min	Inner ring
	Bearing3_4	1515		25 h 15 min	Inner ring
	Bearing3_5	114		1 h 54 min	Outer ring

**Table 7:** List of Autoformer Model structure parameters.

Parameter	Default
encoder input size	1
decoder input size	1
output size	1
seq_len	200
label_len	100
pred_len	50
dimension of model	512
num of heads	8
num of encoder layers	2
num of decoder layers	1

**Table 8:** Comparison between the method proposed in this paper and Informer and Transformer.

Name	Informer	Transformer	Autoformer
MSE	0.5990	0.3812	**0.2638**
MAE	0.5912	0.4247	**0.3506**

**Table 9:** Comparison between the method proposed in this paper and SVR and DBN and CNN and Bi-LSTM.

Name	Proposed Method	SVR	DBN	CNN	Bi-LSTM
MSE	**0.2638**	1.4077	1.1755	0.8639	0.7641
MAE	**0.3506**	1.6880	1.0298	0.8064	0.7125

**Table 10:** Prediction results of the proposed model on different bearings.

Bearing	MSE	MAE	Actual Life	Proposed Method	Double Index
2	0.3826	0.4128	2496	**2508**	2585
5	0.2319	0.2874	114	**118**	98

## Data Availability

The datasets generated during and/or analyzed during the current study are available from the corresponding author on reasonable request.

## Abbreviations

CAE	Convolutional Auto-Encode
ECA	Efficient Channel Attention
RUL	remaining useful life
ConvLSTM	convolutional long and short term memory
WPD	Wavelet Packet Decomposition
CVAE	Conditional Variational Autoencoder
MLP	multilayer perceptual
NLP	natural language processing
GAP	global average pooling
FC	fully connected
MSE	mean square error
MAE	mean absolute error
SVR	Support Vector Regression
DBN	Deep Belief Network
CNN	Convolutional Neural Network
Bi-LSTM	Bidirectional Long Short-Term Memory Network
SVM	Support Vector Machine
xDCNN	X dimension Convolutional Neural Network
$h_{k}$	/
x	/
$x_{i}$	/
$y_{i}$	/
H	Height
W	Width
C	Channels
$L_{10}$	/
γ	/
$\gamma_{t}$	/
$\gamma_{s}$	/
k	/
${\left|\right|}_{odd}$	/
b	/
Fig	Figure
seq_len	Sequence length
label_len	Laber length
pred_len	Predict length
